# A case report of thrombosed varicosities of pubic collateral veins: Ideal treatment strategy and contribution of era imaging technologies in diagnosis

**DOI:** 10.1177/2050313X18757389

**Published:** 2018-02-12

**Authors:** Daniel Paramythiotis, Patroklos Goulas, Petros Bangeas, Argiris Giannopoulos, Kostantinos Kapoulas, Vasileios Rafailidis, Georgios Papadopoulos, Kiriakos Ktenidis, Anna Kalogera-Fountzila, Antonis Michalopoulos

**Affiliations:** 1 1st Propedeutic Surgical Department, AHEPA University Hospital, Aristotle University of Thessaloniki, Thessaloniki, Greece; 2 Vascular Surgery Department, AHEPA University Hospital, Aristotle University of Thessaloniki, Thessaloniki, Greece; 3 Department of Radiology, AHEPA University Hospital of Thessaloniki, Aristotle University of Thessaloniki, Thessaloniki, Greece

**Keywords:** Venous thromboembolism, deep vein thrombosis, collateral circulation, venous recanalization, low-molecular-weight heparin

## Abstract

Collateral circulation is an alternative path occurring in case of venous or artery obstruction. This path may usually develop after primary recanalization. In our case, a 62-year-old woman presented to our Emergency Department complaining about a suprapubic swelling with a cyanotic discoloration of the overlying skin for the past 10 days for which she had been previously prescribed antibiotics. Investigation with ultrasound and contrast-enhanced computed tomography was performed. An imaging study revealed thrombosed pubic varicose collateral veins due to deep vein obstruction and occlusion of the left external iliac vein. The patient was treated with low-molecular-weight heparin, and swelling subsided gradually. Collateral veins of the abdominal wall and over the pubic tubercle are highly predictive of deep venous obstructive disease proximal to the groin level. These collaterals should never be removed, and the patient should be subjected to a diligent laboratory and imaging investigation.

## Introduction

Deep venous thrombosis (DVT) occurs with an incidence of 140–183 per 100,000 patients annually in Europe, while overall thromboembolism (venous thromboembolism (VTE)) is similar to that of stroke.^1^ Rates increase in case of hospitalization, surgery, immobility, trauma, pregnancy, obesity, cancer and a lot of secondary reasons of hypercoagulation.^2^ In the majority of these cases (25%–40%), patients can develop vein recanalization, post-thrombotic syndrome (PTS) and transition to chronic deep venous obstruction. In general, collateral circulation is an anatomical terminology and concerns an alternative path occurring in case of various vascular disorders, such as venous or artery obstruction.^3^ Pubic collateral veins are positive predictive value for deep venous obstruction and usually result of iliac vein obstruction, due to previous DVT and PTS.^3,4^ These varicose collaterals are prone to thrombosis due to their thin wall and vein stasis.^5^ Imaging studies include duplex ultrasound (DUS), computed tomography venography (CTV), magnetic resonance venography (MRV) and classic venography.^3,5,6^ Surgical removal of collateral veins is contraindicated since this could worsen the deep venous circulation, except cases with inflammation and erosion.^2,3^ Treatment of choice is usually conservative, with compression socks, mobilization and anticoagulation.^3^ Although in case of treatment failure, more invasive methods, such as percutaneous transluminal angioplasty and stenting, may have clinical success.^3^ Clinical examination, surgeon suspicion and Clinical–Etiology–Anatomy–Pathophysiology (CEAP) classification of lower extremity chronic venous disorders (C0–C6), is determinant for ideal treatment strategy.^2,3^

## Case report

**Figure 1.**
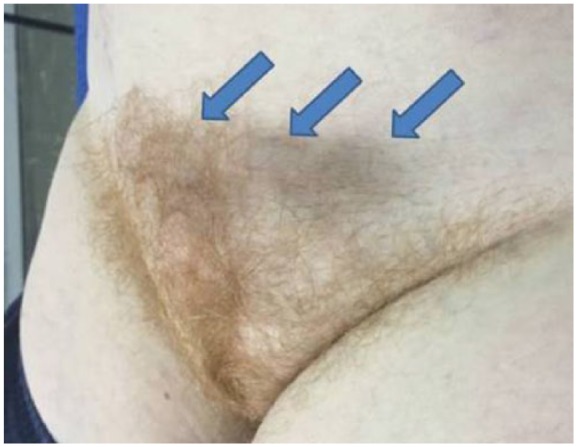
Swelling and bluish discoloration of the skin of lowest hypogastrium due to thrombosed collateral veins (arrow).

**Figure 2.**
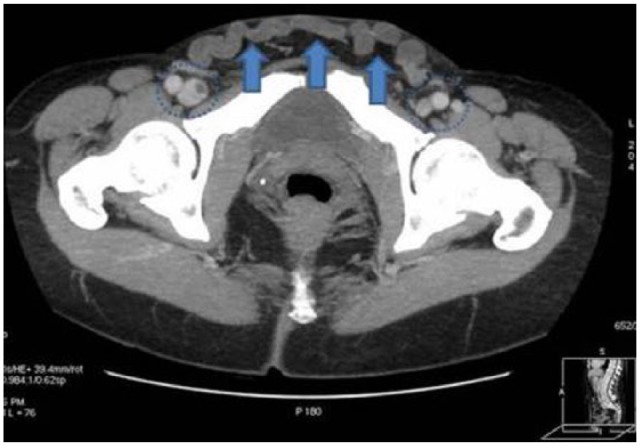
Axial maximum intensity projection (MIP) CT image showing the serpentiform varicose vein connecting the two common femoral veins (circle marker). The venous collateral appears unspecified due to the presence of thrombus which is also seen freely floating inside the right common femoral vein (arrowhead).

**Figure 3.**
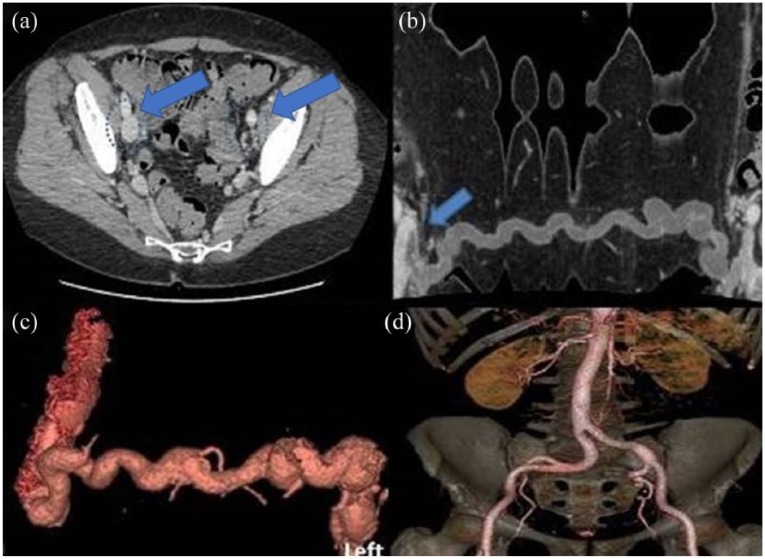
(a) Axial CT image showing the left common femoral vein appearing thread-like in size and unopacified due to suspected chronic thrombosis. Note the asymmetry with the average right size. (b) Curved reconstructed CT image showing the exact course of the collateral vein which is seen connecting the two common femoral veins. Note is made of the freely floating thrombus occupying the whole extent of the collateral and partially the common femoral vein lumen (arrow). (c) Three-dimensional (3D) volume rendering reconstructed CT image showing the collateral vein and the common femoral veins in three dimensions. (d) Virtual reconstruction of arterial phase.

Herein, we present a case of 62-year-old woman submitted to the Emergency Department complaining of a painless swelling in the region of the lowest hypogastrium with a bluish discoloration of the overlying skin. Upon initiation of symptoms (10 days earlier), she was reviewed in a primary healthcare center where she was prescribed antibiotics as septic phlebitis. In palpation, the swelling was reported tender. The patient was afebrile with no signs of inflammation (Figure 1). Patient history was significant for DVT in her left leg 28 years earlier after femoral fracture, with no signs of deep vein insufficiency or PTS. Blood tests revealed D-dimers of 3079 ng/mL with the rest of the parameters being regular. Initially, an ultrasound (US) and computed tomography (CT) of the lesion were performed showing thrombosed veins with no signs of flow within the vessels (Figure 2). The patient was administered low-molecular-weight heparin (LMWH; tinzaparin sodium: 14,000 IU) according to current guidelines treatment. Further investigation with a CT venogram revealed that the suprapubic swelling was thrombosed varicose collateral veins, connecting the two (right and left) femoral veins. The left external iliac vein (EIV) was occluded with signs of chronic thrombosis as its lumen was collapsed (Figure 3(a)–(d)). On the contrary, the right EIV was dilated due to the increased flow in the pubic collaterals. The patient remained under LMWH and was discharged after 7 days with the same medication. Six-month follow-up (FU) duplex scan showed that the thrombus in the pubic collateral veins was partially resolved while the left EIV remained occluded.

## Discussion

Crossover pubic collateral veins are a rare phenomenon but have a profoundly positive predictive value for deep venous obstructive disease proximal to the groin level. This means that there is possibly partial or complete occlusion in the common femoral vein (CFV), the EIV or the common iliac vein (CIV). The most common cause of the venous occlusion is a history of DVT, attributed mainly to non-thrombotic iliac vein compression and pelvic congestion syndrome.^1–3^ The former is also described as May–Thurner syndrome and consists of compression of the left CIV by the overlying right common iliac artery against the vertebral column, whereas the latter is a clinical entity, still not fully understood and sometimes tricky even to diagnose.^2,3^ Pubic crossover veins usually appear as varicosities above the pubic bone and serve as superficial collateral pathways that create an alternative outflow in cases of deep vein obstruction. Their existence is of high importance due to the relief they provided to the patient with deep vein occlusion and their positive predictive value for the diagnosis of the underlying cause. These plexuses are usually formed by swollen superficial external pudenda, superficial epigastric and superficial circumflex iliac veins.^2,4^

Classic imaging studies with US, CT venogram and magnetic resonance imaging (MRI) are enriched with three-dimensional reconstruction images.^5,6^ In cases of deep venous obstruction, these visible collaterals can be the tip of the iceberg, however. Blood outflow can be diverted from the internal iliac vein to the presacral and parametrial plexuses, ipsilateral ascending lumbar vein, ovarian veins or paravertebral plexuses.^2,7^ The formation of suprapubic crossover collaterals is also known as spontaneous Palma shunt because it mimics the well-known Palma operation, that is, a femoro-femoral bypass for deep vein obstruction with contralateral great saphenous vein.^8,9^ Pubic collateral veins should never be operated or removed, even for cosmetic reasons, as this could deteriorate the deep venous insufficiency.^1,9^ Role of venous stenting is under discussion. In a review (40 studies and 2410 limbs stented with FU of 4–48 months), the reported data on stent patency showed an overall primary patency rates between 32% and 98.7%.^10^ In case of severe limb edema, stent angioplasty should be considered. More than one-third of the stented patients showed significant improvement in clinical symptoms (pain, swelling and ulcer).^10,11^ Despite promising results in the literature, complete long-distance occlusions of the iliac axis, as in our case, are not considered an indication for stenting.

## Conclusion

Collateral veins of the abdominal wall and over the pubic tubercle are highly predictive of deep venous obstructive disease proximal to the groin level. One should carefully assess the patient complaining about symptoms of deep venous insufficiency by thorough inspection of the abdominal wall, as this could yield important clinical information. These collaterals should never be removed, and the patient should be subjected to a diligent laboratory and imaging investigation.
